# Interpreter training for medical students: pilot implementation and assessment in a student-run clinic

**DOI:** 10.1186/s12909-016-0760-8

**Published:** 2016-09-29

**Authors:** Jennifer E. L. Diaz, Nydia Ekasumara, Nikhil R. Menon, Edwin Homan, Prashanth Rajarajan, Andrés Ramírez Zamudio, Annie J. Kim, Jason Gruener, Edward Poliandro, David C. Thomas, Yasmin S. Meah, Rainier P. Soriano

**Affiliations:** Department of Medical Education, Icahn School of Medicine at Mount Sinai, New York, NY USA

**Keywords:** Community-oriented, Medicine, Communication skills, Ethics/attitudes, Medical education research

## Abstract

**Background:**

Trained medical interpreters are instrumental to patient satisfaction and quality of care. They are especially important in student-run clinics, where many patients have limited English proficiency. Because student-run clinics have ties to their medical schools, they have access to bilingual students who may volunteer to interpret, but are not necessarily formally trained.

**Methods:**

To study the feasibility and efficacy of leveraging medical student volunteers to improve interpretation services, we performed a pilot study at the student-run clinic at the Icahn School of Medicine at Mount Sinai. In each fall semester in 2012–2015, we implemented a 6-h course providing didactic and interactive training on medical Spanish interpreting techniques and language skills to bilingual students. We then assessed the impact of the course on interpreter abilities.

**Results:**

Participants’ comfort levels, understanding of their roles, and understanding of terminology significantly increased after the course (*p* < 0.05), and these gains remained several months later (*p* < 0.05) and were repeated in an independent cohort. Patients and student clinicians also rated participants highly (averages above 4.5 out of 5) on these measures in real clinical encounters.

**Conclusions:**

These findings suggest that a formal interpreter training course tailored for medical students in the setting of a student-run clinic is feasible and effective. This program for training qualified student interpreters can serve as a model for other settings where medical students serve as interpreters.

**Electronic supplementary material:**

The online version of this article (doi:10.1186/s12909-016-0760-8) contains supplementary material, which is available to authorized users.

## Background

Almost 50 % of US allopathic medical schools operate at least one student-run clinic (SRC). These clinics enhance the training of the future medical workforce [1] and serve as a healthcare safety net by providing free care to a predominantly uninsured minority patient population [2].

A substantial number of patients in SRCs possess limited English proficiency (LEP), a language barrier that often impedes healthcare delivery. An important language in SRCs may be Spanish, as 31 % percent of the US SRC patient population is Hispanic, and nearly 25 % of US Latinos are uninsured, a primary reason that patients attend SRCs [2, 3]. Nearly half of Latinos without citizenship or residency status believe LEP negatively impacts their healthcare [4].

The number of Spanish-speaking patients with LEP attending SRCs and the availability of Spanish-speaking student clinicians caring for them are unknown. Scarcity of student clinicians who speak Spanish fluently enough to provide appropriate care may result in reliance on clinicians with limited Spanish proficiency or untrained ad-hoc interpreters such as patients’ family members or bilingual clinic staff. Untrained interpreters have insufficient medical bilingual skills, use colloquial speech, and make interpreting errors [5, 6], and their use reduces patient and clinician satisfaction [7]. While patients have reported greater comfort when using family members or friends as interpreters instead of professional interpreters [7], ethical issues with this approach include insufficient explanation of important clinical information such as medication adverse effects, and omission of questions about bodily functions, particularly when the ad hoc interpreters are children [8]. Ultimately, patients with LEP who present to non-bilingual clinicians are less satisfied with their care, less likely to receive preventative services, and at greater risk of encountering medical errors [8–11].

One solution to the language barrier, formally training non-fluent student clinicians in SRCs to speak Spanish, is made more difficult by the over-packed medical school curriculum and amount of training necessary for medical Spanish fluency. Alternatively, the use of both in-person and telephone professional interpreters has been shown to facilitate healthcare delivery and increase provider satisfaction [12–15]. However, compared to telephone interpreters, in-person interpreters provide improved non-verbal communication, patient comfort, and patient and physician satisfaction [7, 16] and have been associated with positive benefits in communication, utilization, and clinical outcomes [17]. A training program to prepare already fluent Spanish-speaking students to function as interpreters in the healthcare setting could therefore mitigate this problem in SRCs.

The East Harlem Health Outreach Partnership (EHHOP) is an SRC affiliated with the Icahn School of Medicine at Mount Sinai in East Harlem, one of the most underserved and impoverished neighborhoods of New York City [18, 19]. Because more than half of EHHOP’s patients speak only Spanish, student clinicians continuously struggle with the language barrier. In 2012, we designed a brief, intensive course within the EHHOP Spanish Interpreter Program (ESIP) to train Spanish-fluent medical and graduate students to serve as in-person interpreters. Over a period of 4 years, we assessed the feasibility and efficacy of this pilot program, which may be implemented at other institutions with similar needs.

## Methods

### Course design and needs assessment

The ESIP course design, which was informed by expert consultation and a literature review, incorporated the following qualities of an effective language training program: 1) technique training by a licensed interpreter, 2) vocabulary review, 3) discussion of the needs of the patient population, and 4) a structure that is as interactive as possible. We also analyzed language needs data at our SRC in 53 patient visit records over 4 consecutive clinic days in 2013, and self-reported Spanish proficiency of 156 student clinician volunteer records for 21 clinic days over 5 representative clinic months during 2012–2014.

The ESIP training course was composed of four 90-min modules held in each year 2012–2015 (Table 1). The first two modules were devoted to building interpreting skills, including technical aspects of interpretation and the cultural barriers associated with the interview process. The subsequent two modules were language-intensive and focused on teaching and practicing pertinent medical terminologies, supervised by a professional interpreter or a medical language instructor. In the session on cultural competence and ethics, we emphasized the roles and boundaries of interpreters as patient advocates but not medical experts through group discussion. In the session on difficult interpreting scenarios, we emphasized adhering to fundamental interpreting techniques, such as first-person speaking and clarifying ambiguities, through video tutorials. Students practiced their techniques and module-specific vocabulary via small group role-plays, with participants rotating through patient, physician, and interpreter roles. Based on feedback, we increased interactive practice time following the first year, and this component is emphasized throughout the course (Table 1).

**Table 1:**
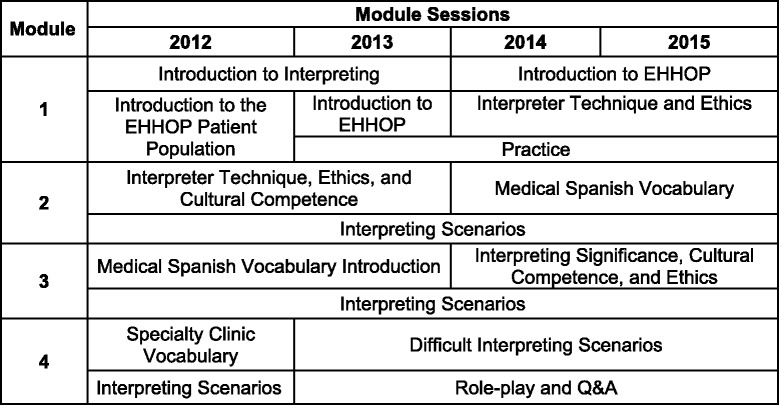
Course outline by year

### Assessments

To evaluate the impact of the program, we obtained assessments of interpreters from three sources: 1) the interpreters themselves, 2) clinicians, and 3) patients. We administered interpreter self-assessments (1) four times: a) *pre-course*: shortly after course registration in each year, b) *post-course*: within 3 weeks of course completion in each year, c) *in-clinic*: immediately following a clinical encounter, and d) *post-clinic*: after having volunteered in clinic. We administered two clinician assessments (2): a) in-clinic, and b) for additional feedback, 4 months after the inaugural interpreters began interpreting in clinic. Patient assessments (3) were administered in-clinic. In-clinic and post-clinic assessments were administered during a 7-month period of active interpreting, 4 to 11 months after the course.

We administered participant self-assessment surveys pre- and post-course using a 5-point Likert scale assessing their overall: 1) comfort with medical interpreting, 2) understanding of their role as an interpreter, 3) familiarity with Spanish terminology of patients from different backgrounds, 4) familiarity with the interpreter’s correct position in the encounter, and 5) comfort interpreting in specialty clinics such as women’s health, mental health, and ophthalmology. Finally, during the 7-month interpreting period, we reevaluated the participants’ post-clinic overall self-assessment of (1) comfort, (2) understanding of their role, and (3) familiarity with terminology (Additional files 1 and 4).

During the 7-month interpreting period following the first 2 years of the course, we administered in-clinic surveys to interpreters, patients, and clinicians, assessing on a 5-point Likert scale the (1) comfort, (2) understanding of role and (3) familiarity with terminology of each interpreter in a specific encounter (Additional file 2). In an additional survey, we asked clinicians to rate on a 5-point Likert scale the ease of use and perceived patient comfort when using live interpreters and/or telephone interpreters (Additional files 3 and 4).

### Statistics

We analyzed the 2012–2013 and 2013–2014 cohorts separately to evaluate whether results would be replicated between cohorts. For unpaired data, we performed a Kruskal-Wallis test followed by selected Student’s t-tests for normal data and selected Wilcoxon-Mann-Whitney (WMW) tests for not normal data. For paired, not normal data, we used a Friedman test followed by selected Wilcoxon-signed-rank (WSR) tests. Data were analyzed using Prism 5 statistical software (GraphPad Software, Inc., La Jolla, CA).

## Results

During the research period, we found that on an average clinic day in our SRC, 63 % (SD = 17 %) (8.5 of 13.3) of patients spoke only Spanish, while only 32 % (SD = 16 %) (2.4 of 7.4) of student-clinicians were proficient in Spanish. Sixty-two students completed the ESIP course in 4 years of its implementation (Table 2).

**Table 2:** Participant demographics

	2012–2013 Cohort	2014–2015 Cohort	Total
	(*n* = 34)	(*n* = 28)	(*n* = 62)
Language proficiency			
Native fluent speakers	23 (68 %)	17 (61 %)	40 (65 %)
Non-native fluent speakers	11 (32 %)	11 (39 %)	22 (35 %)
Training level			
Year 1 MD students	26	17	43 (69 %)
Year 2 MD students	4	1	5 (8 %)
Graduate students	4	9	13 (21 %)
Postbaccalaureate Research Education Program student	0	1	1 (2 %)

**Fig. 1:**
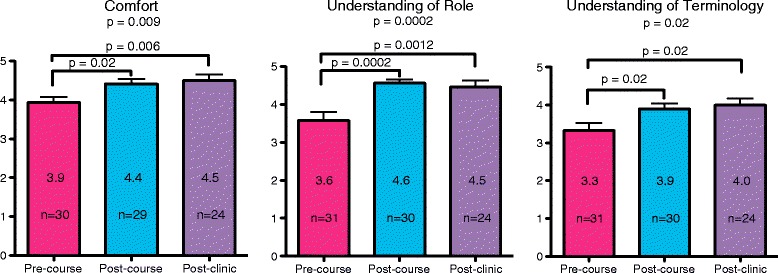
Post-course improvement in self-assessments of course participants. Overall *p* values reflect Kruskal-Wallis tests. Pre- vs. post- course ratings and pre-course vs. post-clinic ratings were tested with either a Student’s *t* test or WMW test as described in methods

**Fig. 2:**
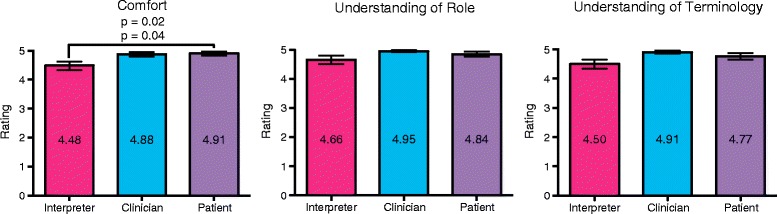
Interpreters are highly rated by patients and clinicians. Overall *p*-value reflects a Friedman test. Interpreter vs. patient ratings were tested with a WSR test as in methods. *n* = 16 interpreters

The 2013–2014 cohort’s self-assessments revealed a significant increase in interpreter comfort, understanding of the interpreter’s role, and familiarity with terminologies used by patients from different cultural backgrounds (Fig. 1; Table 3). Improvements in all three areas persisted several months after completion of the course and after volunteering in clinic (Fig. 1). In addition, we observed a significant increase in interpreters’ understanding of position and interpreters’ comfort in specialty clinic encounters. Most of these results were replicated in the 2014–2015 cohort (Table 3). Both patients and clinicians rated the trained interpreters highly, and we observed a trend that these ratings were higher than the interpreters’ own ratings (Fig. 2). Clinicians rated the ease of use of telephone interpreters and live interpreters similarly but rated perceived patient comfort significantly higher with live interpreters than telephone interpreters (*n* = 30, *p* = 0.003; Additional file 4).

**Table 3:** Participant self-ratings before and after course

	2012–2013 Cohort							2014–2015 Cohort						
	Pre-Course			Post-Course			*p*-value	Pre-Course			Post-Course			*p*-value
	N	Mean	SD	N	Mean	SD		N	Mean	SD	N	Mean	SD	
Comfort	30	3.9	0.78	30	4.4	0.68	**WMW 0.02**	26	3.6	0.85	15	4.1	0.52	**WMW 0.04**
Understanding of Role	31	3.6	1.2	30	4.6	0.50	**WMW 0.0002**	26	3.6	0.98	14	4.8	0.43	**WMW 0.0003**
Familiarity with Terminology	31	3.3	0.03	30	3.9	0.52	**STT 0.02**	26	3.2	1.1	14	3.7	0.83	WMW 0.09
Understanding of Position	29	3.2	1.2	30	4.4	0.56	**STT <0.0001**	26	3.7	1.3	15	4.9	0.35	**WMW 0.0005**
Comfort with Women’s Health	31	3.2	1.0	29	3.8	0.86	**WMW 0.01**	26	3.1	0.77	15	3.7	0.72	**WMW 0.03**
Comfort with Mental Health	31	3.3	0.94	30	4.2	0.61	**STT <0.0001**	26	3.0	0.87	15	3.6	0.63	**WMW 0.04**
Comfort with Ophthalmology	31	3.3	0.97	30	3.9	0.86	**STT 0.02**	26	2.9	0.89	14	3.4	0.76	WMW 0.08

Number of students responding (N) to each survey question, mean and standard deviation (SD) of responses on 5-point Likert scale, and *p*-value of Wilcoxon-Mann-Whitney test (WMW) or Student’s *t* test (STT) of pre- vs. post-course responses as in methods. Significant increases in bold

## Discussion

The discrepancy we have observed at our SRC between the number of Spanish-speaking patients and clinicians highlights the need for language interpreters to ensure patient safety and high quality care. In many institutions, student volunteers are a common source of medical interpreters to fill this language gap, and some bilingual students may serve as informal interpreters in the hospital wards. These experiences serving patients across language and culture barriers may be an important training component for the emerging physician workforce, especially in regions where immigration is on the rise, such as the US [20, 21].

In the limited research to date, medical student interpreters have been found to adopt the role of clinicians, directing the interview, paraphrasing contents, and even serving as patient advocates, a problem we had previously noticed in our SRC [22, 23]. Such actions may impede patient-provider communication, and as the use of untrained interpreters results in lower quality healthcare, it is important to equip these students with proper interpretation skills. While online curricula for this purpose are available [24], formal training has advantages including trained instructors, interactive practice, and a uniform standard of training. We are aware of one program that repurposes the required 40-h training for certified medical interpreters [25] to train medical students, and also requires students to shadow professional interpreters [22]. However, our data suggest that our abbreviated, focused course is sufficient to prepare motivated students to interpret in clinical encounters.

Our results show that a brief 6-h course focused on important interpreting skills facilitated lasting improvements in interpreter comfort, and understanding of terminology and their clinical role. We also observed improved comfort in various clinic settings, and these measures were replicated in an independent cohort. High patient and clinician ratings indicate excellent interpreter performance, similarly to previously reported performance of trained interpreters [7]. These live interpreters may be critical, as we observed increased clinician-perceived patient comfort with trained live interpreters over telephone interpreters.

### Adapting the course to other environments

The course is adaptable to the unique needs of the student participants and patient population. It may be modified for any target language and prior participant training level. It includes time to introduce the specific patient population, addresses ways to effectively advocate for patients in culturally sensitive situations, and trains students to navigate among their roles as interpreter, clinician, and student. For effective adaptations, we stress that practice should be included in all modules.

Our experience shows that an SRC provides fertile ground for launching this curriculum, given significant language needs and an institutional structure that facilitates student involvement and sustainability. In our SRC, formalizing this program improves the quality of interpreting and ensures sufficient interpreter staffing. Fostering collaborations within the medical center facilitates access to qualified teachers. The program may also be utilized to prepare students to interpret during clerkships, and in any clinical environment where medical or pre-medical students wish to serve as interpreters.

### Limitations and future directions

Our study has several limitations. It lacks comparison data of untrained interpreters since they are no longer permitted in our SRC. However, in the future, we hope to compare the performance of our trained interpreters to professional interpreters as well as to patient satisfaction data from encounters that do not require an interpreter. Secondly, we assessed only interpreter performance rather than patient satisfaction, which may be an important surrogate for the quality of patient care, and we hope to investigate this in future studies. In addition, the study lacks assessments by an objective third party. As we found informal role-play was a helpful teaching tool, we hope in the future to use a scored evaluation in formal mock encounters to objectively track retention of skills gained and the success of future changes to the course. Finally, we acknowledge that this pilot study involved a relatively small sample size. We hope other institutions with similar needs will implement training programs for which this course can serve as a model, and replicate our results with larger cohorts.

## Conclusion

Good interpretation skills can facilitate efficient healthcare delivery, ensure patient safety and improve patient care. Students who serve as interpreters face a unique set of challenges, and adequately preparing them to interpret is critical for effective patient-clinician communication. Formal training in second language medical vocabulary and cultural issues could also enhance emerging physician workforce preparedness to serve diverse patient populations. Our pilot program may meet these needs by training medical and graduate students to serve as qualified interpreters, and can potentially serve as a model for teaching hospitals, student-run clinics, and medical centers that also face the challenge of language barriers.

## Additional files

Additional file 1: Supplemental_Survey1.docx. Interpreter Self-Evaluation Survey: Pre- and Post-Course. Survey of course participants evaluating their interpreting ability. Participants took the survey both before and after the course. (DOCX 20 kb) Additional file 2: Supplemental_Survey2.docx. Interpreter Evaluation Survey: Three surveys, one each for clinician, patient, and interpreter, taken immediately following a clinical encounter. The document includes instructions for the interpreter on how to submit the survey. (DOCX 24 kb) Additional file 3: Supplemental_Survey3.docx. Senior Clinician Survey: Survey of student clinicians in our SRC on their use of and satisfaction with interpreters, taken in 2012. (DOCX 98 kb) Additional file 4: Supplementary Methods and Results4.pdf. Supplementary Methods: Survey Administration: Additional details about how the surveys were administered. Supplementary Results: Clinician Feedback: The results of the clinician survey in Additional file 3 show that clinicians perceive higher patient comfort when using a live interpreter. (PDF 61 kb)

## Abbreviations

EHHOP: East Harlem Health Outreach Partnership
ESIP: EHHOP Spanish Interpreter Program
LEP: Limited English proficiency
SRC: Student-run clinic
STT: Student’s *t* test
WMW: Wilcoxon-Mann-Whitney test
WSR: Wilcoxon-signed-rank test
