# Supplementary material for: Interpreter training for medical students: pilot implementation and assessment in a student-run clinic
Source: BMC Med Educ. 2016 Sep 29;16:256. doi: 10.1186/s12909-016-0760-8 (PMC5043630; doi:10.1186/s12909-016-0760-8)
Supplement: Additional file 1: — Supplemental_Survey1.docx. Interpreter Self-Evaluation Survey: Pre- and Post-Course. Survey of course participants evaluating their interpreting ability. Participants took the survey both before and after the course. (DOCX 20 kb) [file 12909_2016_760_MOESM1_ESM.docx]

**Interpreter Self-Evaluation Survey: Pre- and Post-Course**

This research study assesses the effectiveness of interpreters. This survey is anonymous. No one will be able to identify you or your answers. There are no known risks from participating in this study. There is no cost to you to participate. It will take approximately 5 minutes to fill out this survey.

Your participation is voluntary. No compensation will be given. There is no obligation to participate and no consequences for not participating. By choosing to fill out this survey you are volunteering to participate. Your participation ends when you are finished with the survey. You may stop responding and end your participation at any time.

1. What is your confidential interpreter ID number?

2. How comfortable are you translating for EHHOP? (If you have not translated for EHHOP before, how comfortable are you at being a translator?)

not comfortable medium comfort very comfortable

1 2 3 4 5

3. How confident are you with your ability to translate from Spanish to English?

not confident medium confidence very confident

1 2 3 4 5

4. How confident are you with your ability to translate from English to Spanish?

not confident medium confidence very confident

1 2 3 4 5

5. How familiar are you with your role as an interpreter (e.g. whether to translate verbatim what the patient or doctor say, whether you can interject, etc.) in a patient encounter?

not familiar medium familiarity very familiar

1 2 3 4 5

6. How familiar are you with the position of an interpreter (e.g. where to sit in a patient encounter, who to make eye contact with, etc.)?

not familiar medium familiarity very familiar

1 2 3 4 5

7. How confident are you translating for mental health encounters?

not confident medium confidence very confident

1 2 3 4 5

8. How confident are you translating for reproductive health of OBGYN encounters?

not confident medium confidence very confident

1 2 3 4 5

9. How confident are you translating for ophthalmology-related encounters?

not confident medium confidence very confident

1 2 3 4 5

10. How familiar are you with the variance in Spanish terminologies used by EHHOP patients from different cultural backgrounds?

not familiar medium familiarity very familiar

1 2 3 4 5

11. How familiar are you with the East Harlem patient population, with regards to their situations, needs, and health care concerns? *(added in 2015)*

not familiar medium familiarity very familiar

1 2 3 4 5

12. How familiar are you with the clinic flow and components of EHHOP? *(added in 2015)*

not familiar medium familiarity very familiar

1 2 3 4 5

13. Do you think the training course should be mandatory for all EHHOP interpreters? *(only on 2012 post-course survey)*

Yes No
