# Supplementary material for: Interpreter training for medical students: pilot implementation and assessment in a student-run clinic
Source: BMC Med Educ. 2016 Sep 29;16:256. doi: 10.1186/s12909-016-0760-8 (PMC5043630; doi:10.1186/s12909-016-0760-8)
Supplement: Additional file 2: — Supplemental_Survey2.docx. Interpreter Evaluation Survey: Three surveys, one each for clinician, patient, and interpreter, taken immediately following a clinical encounter. The document includes instructions for the interpreter on how to submit the survey. (DOCX 24 kb) [file 12909_2016_760_MOESM2_ESM.docx]

**Interpreter Evaluation Survey**

for Senior Clinicians

This research study assesses the effectiveness of interpreters. This survey is anonymous. No one will be able to identify you or your answers. There are no known risks from participating in this study. There is no cost to you to participate. It will take approximately 5 minutes to fill out this survey.

Your participation is voluntary. No compensation will be given. There is no obligation to participate and no consequences for not participating. By choosing to fill out this survey you are volunteering to participate. Your participation ends when you are finished with the survey. You may stop responding and end your participation at any time.

Please answer the following questions on a scale of 1 to 5, 1 being the worst and 5 being the best.

1. How comfortable did the interpreter seem?

not comfortable medium comfort very comfortable

1 2 3 4 5

2. Did the interpreter demonstrate an appropriate understanding of his/her role? For example, the interpreter:

doesn’t understand medium understanding understands well

1 2 3 4 5

3. How well did the interpreter understand what you mean?

doesn’t understand medium understanding understands well

1 2 3 4 5

Your responses will not be tied to a specific interpreter. Do not use this survey to report specific issues with an interpreter. If you have problems to report, please contact Jenny Diaz directly at [[email]](mailto:andbeonetraveler@gmail.com)

**Put your survey in the envelope and give it and your patient’s survey to the CMs or TS.**

**Encuesta para evaluación del intérprete**

To listen to the survey over the phone, call **9-646-535-2759**. When prompted, press ***** then **1234**. After a few moments, the consent and survey will play in Spanish. **Tell the patient to hang up after hearing the survey.**

Para escuchar la encuesta en español por teléfono, llame **9-646-535-2759**. Cuando le diga, presione ***** y **1234**. Después algunos momentos de inglés, la encuesta comenzará en español. **Cuelgue después de escuchar la encuesta.**

*** * ***

Esta encuesta es parte de una investigación para evaluar la efectividad de los intérpretes. Esta encuesta es anónima. Por favor no escribe su nombre en la encuesta. Nadie podrá identificarlo a usted o sus respuestas. La encuesta no será conectada con un intérprete específicamente. No hay riesgos conocidos si usted decide tomar la encuesta. No hay costo para usted tomar la encuesta. La encuesta tomará más o menos 5 minutos de su tiempo.

La encuesta es voluntaria. Ninguna compensación será dada. No hay obligación participar, ni tampoco consecuencias por no participar. Por llenar la encuesta, usted se pone de acuerdo participar voluntariamente. Su participación termina cuando usted se termine con la encuesta. Puede dejar de llenarla y terminar su participación en cualquier momento.

Por favor responde estas preguntas utilizando una escala de 1 a 5, en que 1 es lo peor y 5 es lo mejor.

1. ¿Qué tan cómodo/a parecía el intérprete?

no cómodo/a medio cómodo/a muy cómodo/a

1 2 3 4 5

2. ¿El interprete demonstra que entiende su papel bien? Por ejemplo, el intérprete:

no lo entiende más o menos entiende bien

1 2 3 4 5

3. ¿Qué tan familiar parecía el intérprete con palabras comunes del país de usted?

no familiar más o menos muy familiar

1 2 3 4 5

**Ponga su encuesta en el sobre y déselo a su doctor.**

**Interpreter Self-Evaluation Survey**

This research study assesses the effectiveness of interpreters. This survey is anonymous. No one will be able to identify you or your answers. There are no known risks from participating in this study. There is no cost to you to participate. It will take approximately 5 minutes to fill out this survey.

Your participation is voluntary. No compensation will be given. There is no obligation to participate and no consequences for not participating. By choosing to fill out this survey you are volunteering to participate. Your participation ends when you are finished with the survey. You may stop responding and end your participation at any time.

**Please answer questions 1-3 with respect to this *specific encounter*.** Please answer on a scale of 1 to 5, 1 being the worst and 5 being the best.

1. How comfortable were you interpreting?

not comfortable medium comfort very comfortable

1 2 3 4 5

2. How familiar did you feel with your role as an interpreter?

not familiar medium familiarity very familiar

1 2 3 4 5

3. How familiar were you with any regional/cultural Spanish terminology used by the patient?

not familiar medium familiarity very familiar

1 2 3 4 5

If you have not answered questions 4-6 on paper before, please **answer them regarding your *overall* interpreting ability.** **You only need to answer questions 4-6 once.**

4. How comfortable are you interpreting for EHHOP?

not comfortable medium comfort very comfortable

1 2 3 4 5

5. How familiar are you with your role as an interpreter (e.g. whether to translate verbatim what the patient or doctor say, whether you can interject, etc.) in a patient encounter?

not familiar medium familiarity very familiar

1 2 3 4 5

6. How familiar are you with the variance in Spanish terminologies used by EHHOP patients from different cultural backgrounds?

not familiar medium familiarity very familiar

1 2 3 4 5

**Put your survey in the envelope and give it to the CMs.**

Instructions to interpreter:

**Write your ID number on each survey.**

After you interpret:

- Give the senior clinician survey to the senior
- Tell the senior that you would like the patient to fill out their survey while waiting for the attending
- Pídele al paciente que llene una pequeña encuesta
- Díle que es completamente **voluntaria** y **anónima**
- Preguntale al paciente si puede leer la encuesta
- If the patient can’t read the survey, the senior can connect them to a recording on the phone.

**Leave the room** and fill out your survey. Gracias!

Instructions to interpreter:

**Write your ID number on each survey.**

After you interpret:

- Give the senior clinician survey to the senior
- Tell the senior that you would like the patient to fill out their survey while waiting for the attending
- Pídele al paciente que llene una pequeña encuesta
- Díle que es completamente **voluntaria** y **anónima**
- Preguntale al paciente si puede leer la encuesta
- If the patient can’t read the survey, the senior can connect them to a recording on the phone.

**Leave the room** and fill out your survey. Gracias!
