# Supplementary material for: Interpreter training for medical students: pilot implementation and assessment in a student-run clinic
Source: BMC Med Educ. 2016 Sep 29;16:256. doi: 10.1186/s12909-016-0760-8 (PMC5043630; doi:10.1186/s12909-016-0760-8)
Supplement: Additional file 3: — Supplemental_Survey3.docx. Senior Clinician Survey: Survey of student clinicians in our SRC on their use of and satisfaction with interpreters, taken in 2012. (DOCX 98 kb) [file 12909_2016_760_MOESM3_ESM.docx]

**Senior Clinician Survey**

This research study assesses the effectiveness of interpreters. This survey is anonymous. No one will be able to identify you or your answers. There are no known risks from participating in this study. There is no cost to you to participate. It will take approximately 5 minutes to fill out this survey.

Your participation is voluntary. No compensation will be given. There is no obligation to participate and no consequences for not participating. By choosing to fill out this survey you are volunteering to participate. Your participation ends when you are finished with the survey. You may stop responding and end your participation at any time.

On a scale of 1 to 5, 5 being the best and 1 being the worst:

1. What was your overall satisfaction with live interpreters in EHHOP last year (schoolyear 2011-2012)?

1 2 3 4 5

2. What was your overall satisfaction with live interpreters in EHHOP over this past semester (fall 2012)?

1 2 3 4 5

3. How easy is Pacific Interpreters (phone) to use in EHHOP?

difficult somewhat easy very easy

1 2 3 4 5

4. How easy is it to use a live interpreter in EHHOP?

difficult somewhat easy very easy

1 2 3 4 5

5. How comfortable do EHHOP patients seem with Pacific Interpreters (phone)?

not comfortable somewhat comfortable very comfortable

1 2 3 4 5

6. How comfortable do EHHOP patients seem with a live interpreter?

not comfortable somewhat comfortable very comfortable

1 2 3 4 5

7. When you want to use a live interpreter in EHHOP, how often is one available?

never sometimes always

1 2 3 4 5

8. How often do you use an ad-hoc interpreter (i.e. a family member or friend who comes in with the patient) in EHHOP?

never sometimes as often as possible

1 2 3 4 5

9. Any comments.
