# Supplementary material for: Interpreter training for medical students: pilot implementation and assessment in a student-run clinic
Source: BMC Med Educ. 2016 Sep 29;16:256. doi: 10.1186/s12909-016-0760-8 (PMC5043630; doi:10.1186/s12909-016-0760-8)
Supplement: Additional file 4: — Supplementary Methods and Results4.pdf. Supplementary Methods: Survey Administration: Additional details about how the surveys were administered. Supplementary Results: Clinician Feedback: The results of the clinician survey in Additional file 3 show that clinicians perceive higher patient comfort when using a live interpreter. (PDF 61 kb) [file 12909_2016_760_MOESM4_ESM.pdf]

Supplementary Methods: Survey Administration

The pre-clinic and post-clinic surveys were administered online. In addition to 5-point Likert scale questions, the post-course survey included free text questions regarding the strengths and weaknesses of the course, including suggestions for future improvement.

The post-clinic survey was administered either online or on paper in the clinic at the participants' convenience. Seventeen interpreters took the survey on paper. Seventeen interpreters took the survey online, two of whom had not yet interpreted in clinic and were therefore excluded. Eight interpreters took both in-clinic and online surveys, and no significant difference was observed between the in-clinic and online surveys in these cases ($p = 0.17$, $p > 0.99$, $p = 0.10$ for comfort, role, and terminology, respectively). Therefore only the in-clinic surveys were included in these cases.

The in-clinic survey was administered on paper immediately following encounters. Qualifying encounters lasted at least 30 minutes and took place in the medical, mental health, women's health, or ophthalmology clinic. A voice recording was used for illiterate patients.

The clinician feedback survey on live and telephone interpreters was administered online.

Supplementary Results: Clinician Feedback

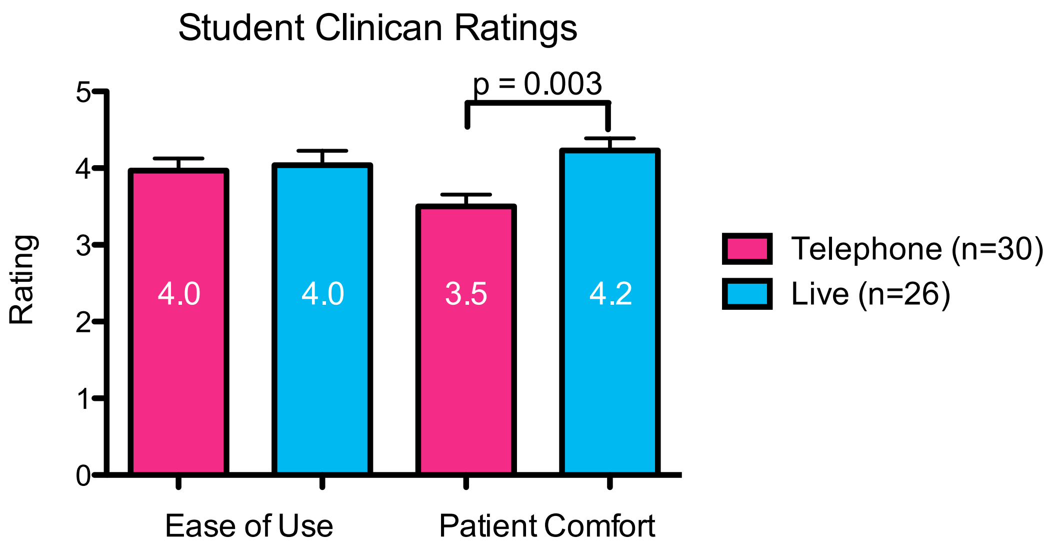

Clinicians perceive higher patient comfort when using a live interpreter. P value reflects a WMW test as in methods.
